# Evaluation of the shear bond strength of a tricalcium silicate-based material to four self-adhering glass ionomer materials: an *in vitro* study

**DOI:** 10.3389/fped.2023.1303005

**Published:** 2023-12-04

**Authors:** Saad BinSaleh, Ayman M. Sulimany, Mannaa K. Aldowsari, Majedah Al-Homaidhi, Nour Alkuait, Lama Almashham, Nada Alghamdi

**Affiliations:** ^1^ Department of Pediatric Dentistry and Orthodontics, College of Dentistry, King Saud University, Riyadh, Saudi Arabia; ^2^ College of Dentistry, King Saud University, Riyadh, Saudi Arabia

**Keywords:** EQUIA Forte HT, NeoMTA 2, resin-modified glass ionomer, shear bond strength, pulpotomy

## Abstract

**Background:**

This study aimed to evaluate and compare the shear bond strength (SBS) of EQUIA Forte HT with that of other restorative materials, including EQUIA Forte, glass ionomer cement (GIC), and resin-modified glass ionomer cement (RMGIC) when bonded to NeoMTA 2.

**Materials and methods:**

A total of 120 holes were created in Teflon molds and filled with NeoMTA 2. The restorative materials were immediately applied using customized silicone molds. The samples were randomly divided into two main groups: one to measure the immediate SBS and the other to measure the delayed SBS. These two main groups were further divided into four subgroups based on the restorative material used: EQUIA Forte HT, EQUIA Forte, GIC, and RMGIC.

**Results:**

The study groups showed statistically significant differences in the mean SBS (*p* < 0.0001). The immediate SBS of the RMGIC group (mean ± SD: 5.43 ± 1.22) was significantly higher than those of the GIC and EQUIA Forte groups, with no significant difference found compared to the SBS of EQUIA Forte HT. In the delayed SBS, both the RMGIC and EQUIA Forte HT groups (4.98 ± 0.67 and 4.93 ± 0.60, respectively) demonstrated significantly higher bond strengths than the GIC and EQUIA Forte groups (3.81 ± 0.57 and 4.2 ± 0.63, respectively). However, there were no statistically significant differences between the RMGIC and EQUIA Forte HT groups or between the GIC and EQUIA Forte groups.

**Conclusion:**

Based on our findings, EQUIA Forte HT has shown promising outcomes when used as a restorative material following pulpotomies, with results comparable to those of RMGIC.

## 1. Introduction

Glass ionomer cement (GIC) materials have a wide range of applications in pediatric and restorative dentistry due to their ability to release fluoride over an extended period (1, 2). However, they are vulnerable to fracture due to their poor flexural strength and fatigue properties (3).

To circumvent these disadvantages, resin-modified glass ionomer cement (RMGIC) was developed. However, RMGIC has a mechanical strength that is inferior to that of composite resin materials and is not considered a permanent restorative material. Furthermore, it has the disadvantage of polymerization shrinkage due to its resin content. To overcome these mechanical limitations, EQUIA Forte was introduced as a long-term restorative alternative.

EQUIA Forte is a self-curing, resin-free, highly viscous GIC that releases fluoride (F^−^) and calcium ions (Ca^2+^) (4). It is a bulk-fill glass hybrid (GH) reinforced with ultrafine reactive glass particles. To strengthen the cross-linking of the matrix and enhance the material's flexural strength, EQUIA Forte has also been reinforced with secondary silicate particles of smaller size and higher reactivity, along with acrylic acid molecules of elevated molecular weight. The application of a resin coat to these restorations is allegedly meant to enhance both their wear resistance and esthetic appearance (5).

To further enhance the aesthetic properties, EQUIA Forte HT (GC, Tokyo, Japan) was released in 2019, featuring increased translucency (6). This restorative material exhibits a refined smaller particle size distribution in comparison to its precursor, EQUIA Forte. This refinement contributed to the enhancement of both flexural and compressive strength, resulting in improved matrix loading (7).

Several studies have been conducted on EQUIA Forte HT, demonstrating its reliability as a restorative material with good clinical performance in both primary and permanent teeth (5, 8). Kutuk et al. conducted a study on EQUIA Forte HT in stress-bearing Class II restorations in pediatric patients and concluded that it offers more strength and superior aesthetics compared to a microhybrid composite (G-aenial Posterior, GC Corp., Tokyo, Japan) for both primary and permanent teeth (5).

To date, the application of the relatively newly introduced EQUIA Forte HT to the novel bioceramic material NeoMTA 2 (NuSmile, Houston, TX, USA) has not been studied for its use in primary teeth after pulpotomies. Therefore, this study aimed to evaluate and compare the shear bond strength (SBS) of EQUIA Forte HT with that of other restorative materials—namely, EQUIA Forte, GIC, and RMGIC—when applied to NeoMTA 2.

## 2. Materials and methods

### 2.1. Ethical clearance

Ethical clearance for this study was obtained from the institutional review board (No. E-22-7160), and the College of Dentistry Research Center (CDRC No. IR 043).

### 2.2. Sample size calculation

The sample size was calculated from the previously available literature with a 95% confidence interval and 80% power of the study. For an *α* value of 0.05, an effect size of 0.45, and a power of 0.95, the total sample size should be at least 120.

### 2.3. Study design

A total of 120 holes were created in Teflon molds, each measuring 4 mm in diameter and 2 mm in depth, filled with NeoMTA 2. Next, the surface was smoothened using a plastic filling instrument (PF13) to prepare the samples for bonding. A universal adhesive (Single Bond Universal 3M, ESPE, St. Paul, MN, USA) was applied to the surface of the bioceramic material using a rubbing motion for 20 s. The adhesive was subsequently subjected to air drying for 5 s and a light-curing process for 10 s.

To apply the restorative material, a customized silicone mold with a diameter of 3 mm and a thickness of 2 mm was fabricated for use in the bonding procedure. The mold was placed at the center of the NeoMTA 2 layer. The restorative materials were applied immediately after the placement of NeoMTA 2 according to the manufacturer's instructions (Table 1).

**Table 1:** Materials used in the study (name, company, chemical composition, and manufacturer's instructions).

Material	Company	Chemical composition	Manufacturer's instructions
EQUIA Forte HT Fil shade A2	GC Corp., Tokyo, Japan	○ Powder: 95% strontium fluoroaluminosilicate glass, 5% polyacrylic acid ○ Liquid: 40% aqueous polyacrylic acid ○ EQUIA Forte Coat: 40%–50% methyl methacrylate, 10%–15% colloidal silica, 0.09% camphorquinone, 30%–40% urethane methacrylate, 1%–5% phosphoric ester monomer	○ Shake or tap the capsule to loosen the powder. ○ Depress the plunger and hold it down for 2 s. ○ Mix for 10 s. ○ Dispense within 10 s. ○ Pack, contour, and ensure that the restorative material is fully set. ○ Finish the restoration by applying EQUIA Forte HT Coat. ○ Light cure for 20 s.
EQUIA Forte Fil shade A2	GC Corp., Tokyo, Japan	○ Diurethane and methacrylate-based monomers with a modified polyacrylic acid and polybutadiene-modified diurethane dimethacrylate	○ Apply petroleum jelly inside the matrix. ○ Apply the GC Cavity Conditioner for 10 s. ○ Mix for 10 s. ○ Dispense within 10 s. ○ Finish the restoration by applying the EQUIA Coat. ○ Light cure for 20 s.
GIC Fuji^TM^ II	GC Corp., Tokyo, Japan	○ Powder: fluoroaluminosilicate glass ○ Liquid: acrylic acid, maleic acid, tartaric acid, water	○ Add 1 level scoop of powder to 1 drop of liquid. ○ Mix the required amount of cement. ○ Form the contour during the first 2 min of setting.
GC Fuji II LC® resin-reinforced glass ionomer Fil shade A2	GC Corp., Tokyo, Japan	○ 25%–50% 2-hydroxyethylmethacrylate (HEMA) ○ 5%–10% polybasic carboxylic acid ○ 1%–5% urethane dimethacrylate (UDMA) ○ 1%–5% dimethacrylate	○ Shake the capsule or tap its side on a hard surface to loosen the powder. ○ Mix for 10 s. ○ Form the contour and place a matrix if required. ○ Light cure for 20 s.
NeoMTA 2	NuSmile Inc., Houston, TX, USA	○ Powder: tricalcium silicate (Ca_3_SiO_5_), dicalcium silicate (Ca_2_SiO_4_), tantalum oxide (Ta_2_O_5_), and minor amounts of calcium sulfate (CaSO_4_) and tricalcium aluminate (Ca_3_Al_2_O_6_) ○ Liquid: water (H_2_O) and proprietary polymers different from the above	○ Add 1 level scoop of powder. ○ Dispense a drop of gel. ○ Integrate thoroughly. ○ Spread NeoMTA. ○ Complete the restoration immediately.
Tetric N Ceram Bond	Ivoclar Vivadent Inc. Principality, Liechtenstein.	○ BisGMA (25%–50%), water and ethanol (10%–25%), 2-hydroxyethyl methacrylate (HEMA) (10%–25%), phosphonic acid methacrylate (MDP) (10%–25%), diphenyl(2,4,6-trimethylbenzoyl)phosphine oxide (1%–2.5%), urethane dimethacrylate (0.3%–10%)	○ Apply a coat of adhesive and agitate for 20 s. ○ Disperse the adhesive with compressed air until a glossy, firm layer is obtained. ○ Light-cure for 10 s.

An LED light-curing device (Bluephase G2, Ivoclar Vivadent, Schaan, Liechtenstein) was used to cure all the samples. After that, the samples were divided equally into two main groups: one to measure the immediate SBS and the other to measure the delayed SBS.

Before performing the SBS tests, all the samples were preserved in artificial saliva and stored in an incubator (GI2 So-Low Cincinnati, OH, USA) at 37 °C and 100% humidity for 24 h. Next, only the samples in the delayed restorative group were loaded in a thermocycling machine (Huber, SD-Mechatronik-Thermocyclerr, Germany) at 5 °C for 2,000 cycles of thermocycling and 55 °C for 5,000 cycles of thermocycling to mimic 6 months of physiological use.

### 2.4. SBS test

The SBS of each sample was determined using a universal testing machine (Instron 5965, Instron, England) with a knife-edged rod moving at a crosshead speed of 0.5 mm/min (Supplementary Material Figure S1). The SBS was calculated in MPa using the following formula: stress (MPa) = force^2^ (N)/bonding area (mm^2^).

In the case of the immediate SBS group, after 24 h, the samples were mounted in the universal testing machine with the crosshead perpendicular and flush to the interface of the restoration and the bioceramic material (Supplementary Material Figure S2). For the delayed SBS group, the SBS test was performed after 7 days in the same manner as for the immediate SBS group.

### 2.5. Mode of failure

To discover the fracture pattern after the SBS test, the samples' surfaces were examined using a digital microscope (HIROX, KH-7700, Digital microscope system, Tokyo, Japan).

The failure modes were categorized as follows: (1) adhesive failure: failure between the NeoMTA 2 layer and the restorative material; (2) cohesive failure type 1: cohesive failure within the NeoMTA 2 layer; (3) cohesive failure type 2: cohesive failure within the restorative material; and (4) mixed failure: both adhesive and cohesive failures.

### 2.6. Statistical analysis

The immediate SBS and SBS after the aging process of NeoMTA 2 combined with the four types of restorative materials were reported as the mean ± standard deviation (SD). The mean SBS of each group was determined using a one-way analysis of variance (ANOVA) followed by Tukey's multiple comparison test to assess the differences between the groups. The significance of the difference between the immediate and delayed SBS for each material was calculated using the Student's *t*-test. The data were analyzed using SAS 9.4 (SAS Institute, Inc., Cary, NC, USA). A *p*-value of <0.05 was considered statistically significant.

## 3. Results

Table 2 shows the mean immediate SBSs of the self-adhering glass ionomer materials on NeoMTA 2. There were statistically significant differences between the mean SBSs of some of the study groups (*p* = 0.005). The SBS of the RMGIC group (5.43 ± 1.22) was significantly higher than that of the GIC and EQUIA Forte groups (4.32 ± 1.24 and 4.2 ± 0.63, respectively). However, there was no statistically significant difference between the RMGIC group and the EQUIA Forte HT group.

**Table 2:** Immediate shear bond strength of the different study materials.

Group	Mean SBS (MPa)	SD	*p*-Value
RMGIC (g4)	5.43^a^	1.22	0.005
GIC (g3)	4.32^b^	1.24	
EQUIA Forte (g2)	4.20^b^	0.63	
EQUIA Forte HT (g1)	5.14^a,b^	1.08	

Different numbered letters (g1, g2, g3 and g4) indicate different sample groups.

Different superscript lowercase letters indicate significant differences between materials *p* ≤ 0.005.

The mean SBSs of the self-adhering glass ionomer materials to NeoMTA 2 after the aging process are presented in Table 3. There was a statistically significant difference between the mean SBSs of some of the study groups (*p* < 0.0001). The SBSs of the RMGIC and EQUIA Forte groups (4.98 ± 0.67 and 4.93 ± 0.60, respectively) were significantly higher than those of the GIC and EQUIA Forte groups (3.81 ± 0.57 and 4.2 ± 0.63, respectively). However, there was no statistically significant difference between the RMGIC and EQUIA Forte HT groups or between the GIC and EQUIA Forte groups.

**Table 3:** Shear bond strength of the different study materials after the aging process.

Group	Mean SBS (MPa)	SD	*p*-Value
RMGIC (g4)	4.98^a^	0.67	<0.0001
GIC (g3)	3.81^b^	0.57	
EQUIA Forte (g2)	3.94^b^	0.77	
EQUIA Forte HT (g1)	4.93^a^	0.60	

Different numbered letters (g1, g2, g3 and g4) indicate different sample groups.

Different superscript lowercase letters indicate significant differences between materials.

Table 4 presents the mean SBSs of the different study materials at different times. Overall, the immediate SBS was higher than the SBS after the aging process among all groups, although the differences were not statistically significant.

**Table 4:** Mean shear bond strength of the different study materials at different times.

Group	Immediate mean SBS (MPa) (mean ± SD)	Delayed Mean SBS (MPa) (mean ± SD)	*p*-Value
RMGIC	5.43 ± 1.22	4.98 ± 0.67	0.35
GIC	4.32 ± 1.24	3.81 ± 0.57	0.66
EQUIA Forte	4.20^ ^± 0.63	3.94 ± 0.77	0.26
EQUIA Forte HT	5.14^ ^± 1.08	4.93 ± 0.60	0.74

The types of failure modes in the immediate and delayed SBS groups are represented in Supplementary Material Figure S4, S5, respectively. In the immediate SBS groups, EQUIA Forte HT showed a combination (cohesive–adhesive) failure rate of 53.3%, while Equia Forte and GIC had adhesive failure rates of 93.3% and 86.6%, respectively. Finally, RMGIC showed a cohesive failure rate within the NeoMTA 2 layer of 93.3%.

In the delayed SBS groups, cohesive failure within the NeoMTA 2 layer was the most common form of failure found in the EQUIA Forte HT (66.6%), EQUIA Forte (53.3%), and RMGIC (73.3%) groups. However, adhesive failure was predominant in the GIC group (80%).

## 4. Discussion

Within the limitations of the current study, the null hypothesis that there was no significant difference between the SBS of EQUIA Forte HT and that of other restorative materials—namely, EQUIA Forte, GIC, and RMGIC—when combined with NeoMTA 2 was rejected. The results of the present study regarding both the immediate and delayed SBS revealed a significantly higher mean SBS for EQUIA Forte HT compared to GIC and EQUIA Forte. However, RMGIC showed the highest mean SBS, which is consistent with a previous study, which found that the immediate placement of RMGIC on ProRoot MTA showed the highest SBS (7.18 MPa), followed by NeoMTA 2 (4.15 MPa) (9). Al-homaidhi et al. reported that the SBS of RMGI immediately placed over NeoMTA 2 was statistically high (10), which is consistent with the results of this study.

In another recent study conducted by El-Refai et al. in 2023 (11), the delayed SBSs of four different restorative materials (Fuji II LC, EQUIA Forte Fil, Cention N, and Venus Bulk Fil) applied to a pre-mixed bioceramic material (NeoPutty) were calculated. The findings indicated that the mean SBS for the RMGIC (Fuji II LC) was high (18.33 ± 2.29 MPa), which is consistent with the results of our study. However, it was demonstrated that EQUIA Forte exhibited the lowest mean SBS (7.07 ± 1.06 MPa) of all materials. When comparing the findings of prior research, it can be observed that NeoPutty exhibits similarities to NeoMTA 2 in terms of its primary compositional elements. Similarly, EQUIA Forte can be regarded as comparable to EQUIA Forte HT (7, 12). However, it is crucial to consider a significant aspect of the methodology employed in the previous literature: the delayed application time of the restorative material.

Previous studies (13–15) have provided evidence in favor of delayed restoration following the placement of mineral trioxide aggregate (MTA). It has been claimed that the water sorption from freshly mixed MTA could be attributed to the presence of GIC, resulting in inadequate hydration of MTA and the presence of significant porosity, the interface junction between the glass ionomer and MTA exhibited a notable presence of microcracking, leading to the separation of the two materials and the subsequent degradation of the adhesive junction (11). However, additional research (16–18) has provided support for the immediate application of the restorative material directly over the MTA, taking the advantage of short initial setting time of 14 min at 37 °C, thereby aligning with the real clinical scenario.

A study conducted by Nandini et al. (16) showed that conventional GIC may be applied over a partially set MTA in a single visit. The authors also observed that the setting process of MTA remained unaffected beneath the GIC layer. Another study conducted by Palma et al. (19) yielded results comparable to those of our study: the researchers concluded that the use of Biodentine and the immediate placement of the final restoration yielded the highest average SBS (4.44 MPa). Furthermore, Alqahtani et al. (9) stated that the immediate application of the Fuji II LC resin-modified glass ionomer on ProRoot MTA exhibited significantly higher SBS compared to delayed application.

Regarding the modification of the MTA surface before the application of restorative materials, previous studies (14, 20, 21) have demonstrated that the process of acid etching when performed as a separate step, can lead to the degradation of the MTA surface. This degradation is characterized by a reduction in cohesive strength, a decrease in microhardness, a decline in compressive strength, and the formation of an amorphous gel-like surface structure.

Furthermore, it is imperative to prioritize the simplification of dental procedures and the reduction of working time when addressing pediatric patients. Based on this fact, it can be inferred that employing a universal bonding agent without performing a distinct pre-etching procedure would be the more favorable approach. Furthermore, the selection of the 3M Single Bond Universal Adhesive for the present study was predicated upon its pH value of 2.7. Based on this characteristic, it can be categorized as an ultra-mild etching system, thereby enabling the generation of appropriate surface micro-irregularities on the NeoMTA 2 surface while maintaining the integrity of the surface crystals, which is essential for establishing a strong adhesive junction (22).

In the present study, EQUIA Forte HT exhibited the second highest SBS among all the materials. When a conventional GIC is applied on top of MTA, two potential reactions may occur at the interface. First, the carboxylate groups (COO^–^) of the polyacrylic acid within the GIC may interact with the calcium ions present in the MTA, resulting in the formation of calcium salts. Second, the silicate hydrate gel of the MTA can undergo condensation with the silicate hydrate gel of the GIC, leading to the generation of by-products (16). Rodríguez-Lozano et al. (23) demonstrated that NeoMTA 2 exhibits a greater release of calcium ions compared to alternative bioceramic materials, such as NeoMTA Plus and Bio-C Repair, suggesting the theoretical existence of a strong bond between EQUIA Forte HT and NeoMTA 2. However, the lack of calcium, which is substituted by strontium in EQUIA Forte HT, could be a cause of the weak bonding to NeoMTA 2, which resulted in a lower mean SBS than in conventional RMGIC. Nicholson et al. (24) also stated that strontium functions as a cement-forming ion. However, the addition of strontium oxide powder to GIC did not significantly enhance its properties (24).

EQUIA Forte HT is a high-viscosity GIC that does not contain any resinous constituents. As a result, the formation of strong chemical bonds with the resinous component of the universal adhesive is not feasible, suggesting potential limitations in the capacity for chemical bonding with 10-methacryloyloxydecyl dihydrogen phosphate (10-MDP) molecules. The main mechanism of bonding in EQUIA Forte HT is likely the micromechanical interlocking facilitated by etching the surface of NeoMTA 2 using the ultra-mild universal adhesive. Nevertheless, the resulting micromechanical interlocking may not have had major effects. This is primarily due to the high viscosity of EQUIA Forte, which may have impeded its flow and penetration into the micro-irregularities of the NeoMTA 2 surface (11).

This suggests that the primary chemical interaction occurs between the silicate hydrate gel of the MTA and the silicate hydrate gel of the GIC. It should be noted that there is a lack of literature on the chemical interactions at the interface between EQUIA Forte HT and NeoMTA 2. Tsuzuki et al. found that the early stages of the GIC-setting reaction comprised both endothermic and exothermic reactions, confirming the occurrence of reactions other than carboxylate formation (25).

In 2021, Duman et al. compared the SBSs of Medcem Pure Portland Cement, Medcem MTA, and NeoMTA to those of compomer, RMGIC, EQUIA Forte HT, and Cention N (26). They found that EQUIA Forte HT showed the highest SBS along with Medcem Pure Portland Cement, while the lowest SBSs of all groups were yielded by EQUIA Forte HT and NeoMTA.

In the previous study done by Duman et al.2021, the samples were loaded with different biomaterials and held at 37 °C for 4 h in distilled water, as it was believed that a moist environment is necessary to ensure the appropriate setting of MTA. However, this technique proved ineffective due to the lack of moisture control, resulting in heightened porosity and solubility, which ultimately compromised the material's strength (27). Therefore, preserving the samples for 4 h might not be favorable for the experiment, which is why this step was not performed in our study.

Moreover, comparing the findings of different research studies is challenging due to the variability of multiple relevant factors, including the specific characteristics of the restorative materials, adhesive systems, technical application methods, and restoration application time used. Such comparisons are further complicated by variations in experimental methods, such as the rate of load application and the magnitude of the maximum load employed to assess the SBS.

Overall, the immediate SBS was higher than the SBS after the aging process among all groups in our study, although the difference was not statistically significant. Concerning the failure modes, the examination demonstrated that the two most common failure modes for EQUIA Forte HT were cohesive failure within the bioceramic material (Neo MTA 2) or combination (cohesive–adhesive) failure.

Cohesive failure indicates that the materials have reached maximum adhesive strength; therefore, it is a preferred mode of fracture. In the present study, the immediate SBS of EQUIA Forte HT showed a 53.3% combination (cohesive–adhesive) failure rate and a 66.6% cohesive failure rate within NeoMTA 2 in the delayed SBS group. The results are somewhat consistent with previously conducted studies on MTA and premixed bioceramics, which showed more cohesive and mixed cohesive–adhesive failures (9, 10).

Davis et al. explained the intricate nature of mixed-mode failures (28). These failures pose challenges in terms of interpretation, as it is uncertain whether the bond failure was the cause or the consequence of the crash. Quantifying the degree of bond degradation is difficult, as it is inherently subjective and cannot be measured precisely. Consequently, the terminology employed to describe the extent of degradation is limited to subjective terms, such as “moderate” or “predominant.” The sole ascertainable fact is that the bond strength decreased compared to its initial value. Regarding the impact of thermocycling on the SBS, Arıc et al. determined that the mean SBS of RMGIC decreased following this process (29).

*In vitro*, studies ignore the presence of dentin and thus do not fully reflect real clinical settings in which three interfaces can be identified: the interface between the bioceramic and dentin, the interface between the restorative material and dentin, and the interface between the bioceramic and the restorative material. This study assessed only the third interface. Therefore, further clinical trials are needed, particularly in the presence of saliva contamination. Additionally, only immediate placement was assessed in this study, highlighting the pressing need for future studies to assess the delayed application time of the restorative material.

## 5. Conclusion

Following conclusions are drawn. First, this study can aid clinicians in selecting the optimal material for clinical applications. Based on our results, EQUIA Forte HT has shown promising outcomes when used as a restorative material following MTA pulpotomies, with results comparable to those of RMGIC. Second, we showed that the immediate SBS was higher than the SBS after the aging process among all the studied groups. However, these differences were not statistically significant.

## Data Availability

The raw data supporting the conclusions of this article will be made available by the authors, without undue reservation.
