# Supplementary material for: Evaluation of the shear bond strength of a tricalcium silicate-based material to four self-adhering glass ionomer materials: an in vitro study
Source: Front Pediatr. 2023 Dec 4;11:1303005. doi: 10.3389/fped.2023.1303005 (PMC10726004; doi:10.3389/fped.2023.1303005)
Supplement: Supplementary file 1 [file Table1.docx]

Supplementary Material

# Supplementary Figures

## Supplementary Figures

**
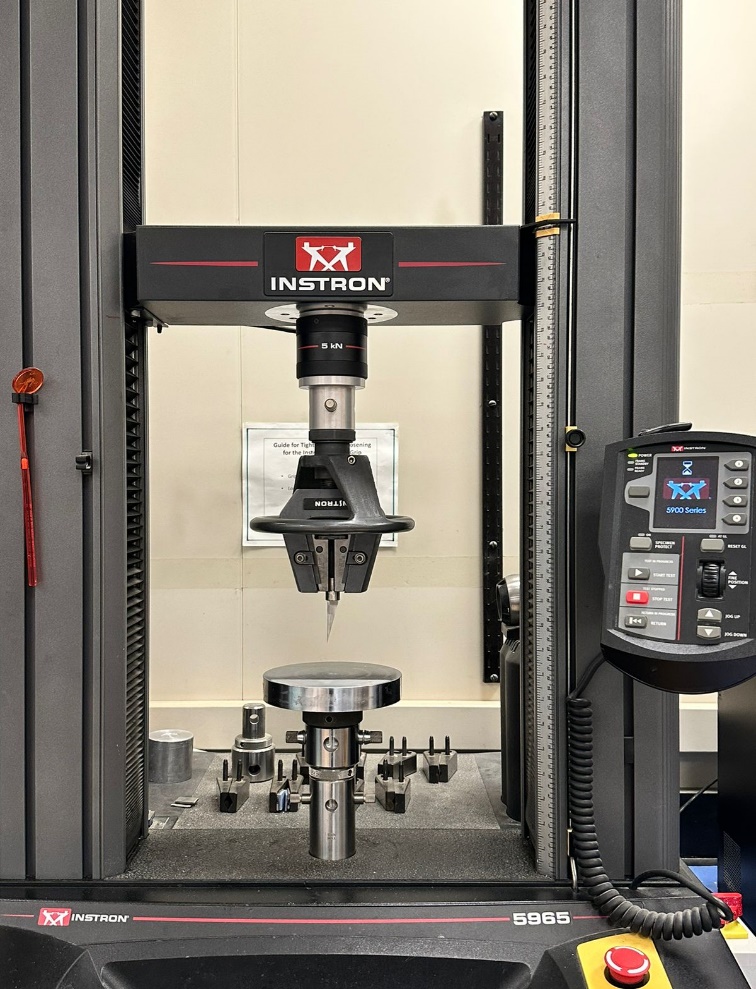
**

**Supplementary Figure 1** Universal testing machine (Instron5965, Instron, England)

**
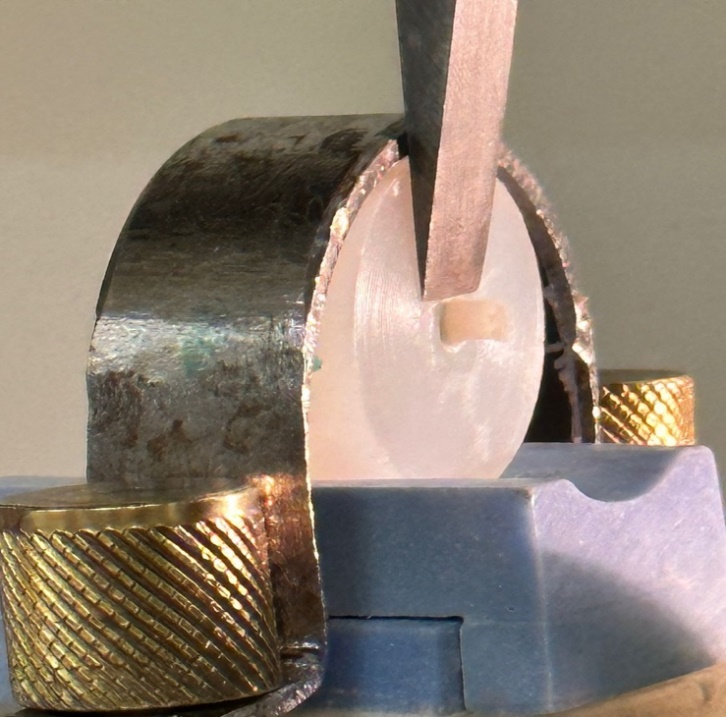
**

**Supplementary Figure 2** SBS testing at the interface between the NeoMTA 2 and the restorative material


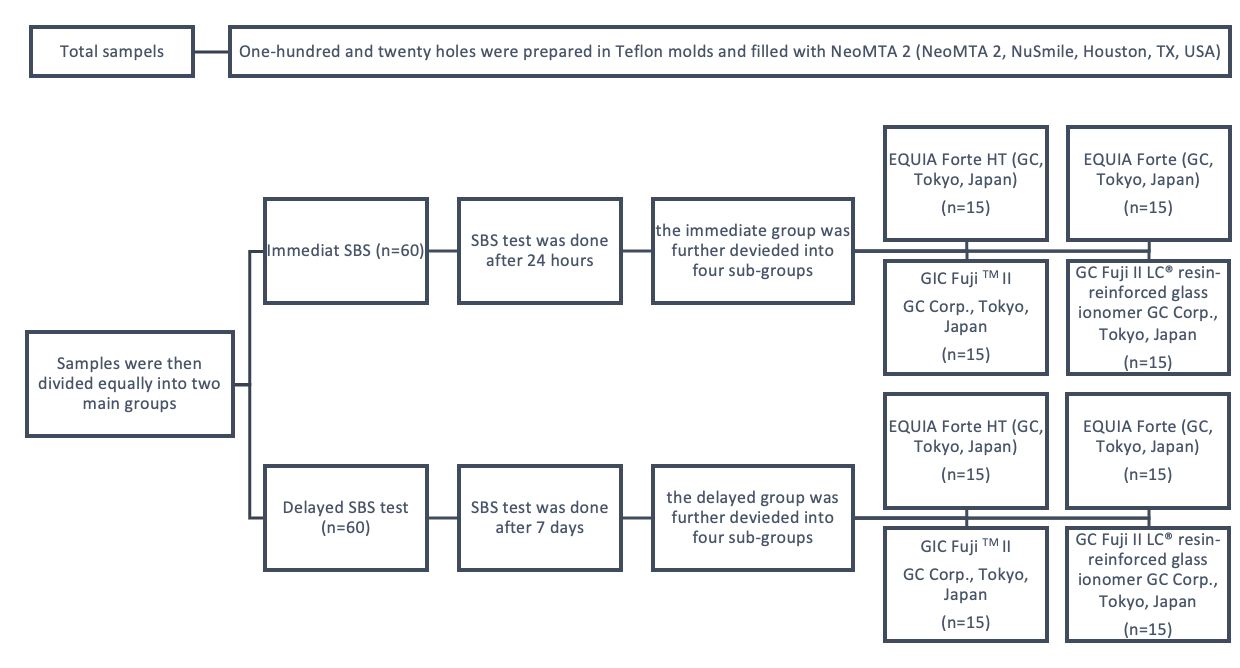


**Supplementary Figure 3:** Flow chart showing the groups’ distribution based on the time of testing the SBS

**
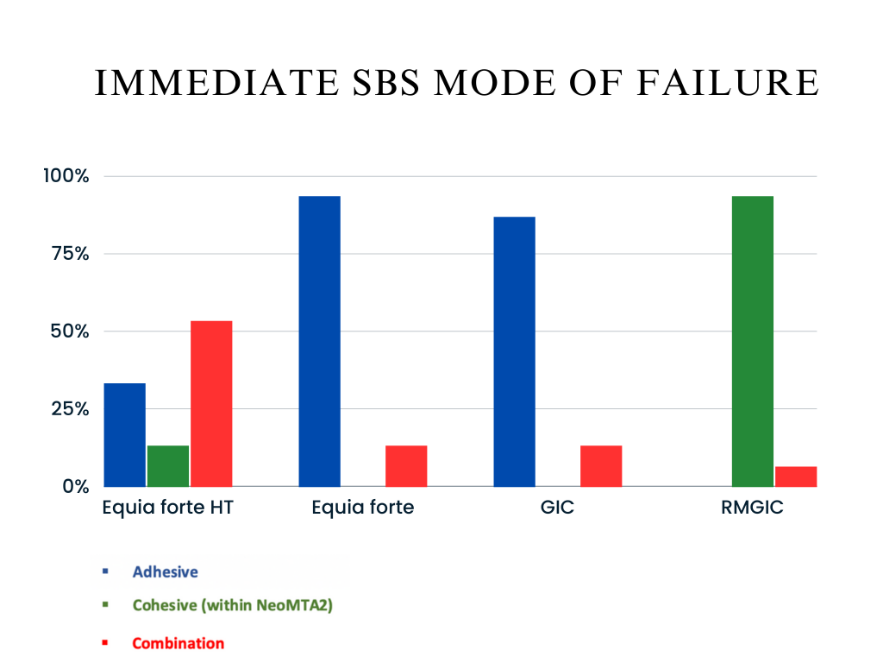
**

**Supplementary Figure 4:** Bar chart representing the failure modes of the immediate SBS groups.

**
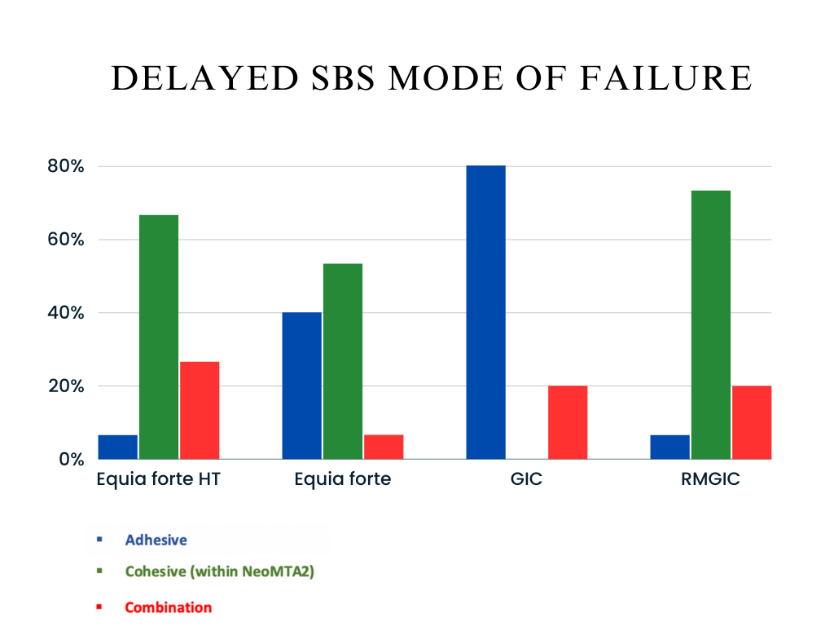
**

**Supplementary Figure 5**: Bar chart representing the failure modes of the delayed SBS groups.

**Supplementary**
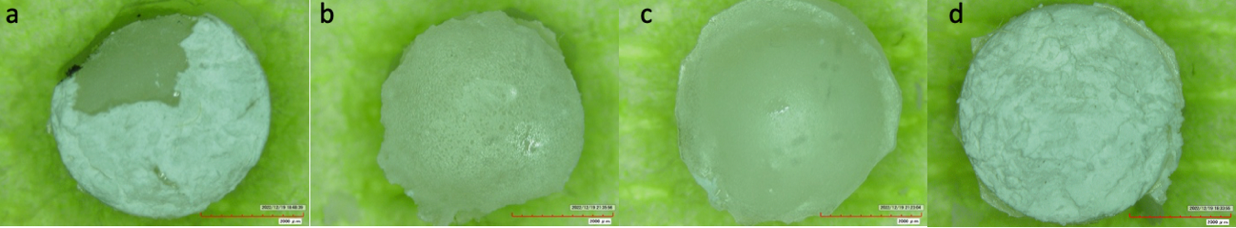
**Figure 6:** The micrographs of the specimens were examined after 24 hours using a digital microscope (HIROX, KH-7700, Digital microscope system, Tokyo, Japan): (a) EQUIA Forte HT, (b) EQUIA Forte, (c) GIC, and (d) RMGIC.


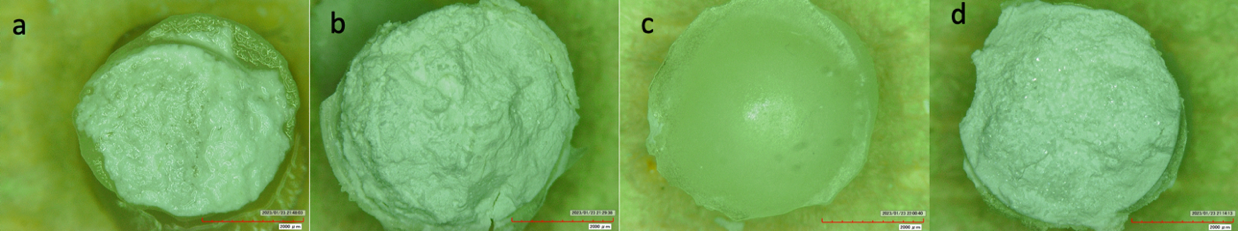


**Supplementary Figure 7:** The micrographs of the specimens were examined after the aging process (7 days) using a digital microscope (HIROX, KH-7700, Digital microscope system, Tokyo, Japan): (a) EQUIA Forte HT, (b) EQUIA Forte, (c) GIC, and (d) RMGIC.
